# Supplementary material for: Empirical Bayes factors for common hypothesis tests
Source: PLoS One. 2024 Feb 22;19(2):e0297874. doi: 10.1371/journal.pone.0297874 (PMC10883543; doi:10.1371/journal.pone.0297874)
Supplement: Attachment — Submitted filename: Response to reviews.docx [file pone.0297874.s002.docx]

# Response to reviews

I appreciate the reviewers’ feedback. My responses are below in the indented paragraphs. Line numbers refer to the change-tracked document.

Reviewer #1: Under review is the proposal for an "empirical Bayes factor". The derivation begins by considering the "bias" in the posterior marginal likelihood, which the empirical Bayes factor involves correcting for. It was unclear to me why the formula for b_H(x) was designated as "bias" that should be corrected.

This was explained at line 110, “The posterior marginal likelihood may be biased, in the sense of being systematically different from a marginal likelihood with the prior for θ supplied independently of X.” However, I agree that my previous definition of b_H(X) did not match this description. I have given a new definition at line 115 which switches the order of X and Y in taking expectations; this better reflects the idea expressed above.

Specifically, a particular realisation of replicate data Y is taken to provide an independent prior, and the bias is now defined as the expected difference (over observed data X) between the log posterior marginal likelihood and the log marginal likelihood with prior learned from y. We then take the expectation of this bias over Y to arrive at the same results as before.

As for why this bias should be corrected, it was argued at lines 50-58 that it is to obtain a measure that is interpretable on the same scale as a proper Bayes factor. See further below.

It was not clear to me why the averaged behavior across draws of Y should be of first-order importance in a Bayesian analysis. It seems quite standard in Bayesian analysis for quantities to be biased (as generally Bayesian estimates are biased) and it would be unclear why in general such bias would need to be corrected for. It would seem necessary to set up a formal justification for this, and other steps in the statistical analysis.

A simple answer is that this is not a proper Bayesian analysis. (The discussions of the papers by O’Hagan, Aitkin and Bernardo [19, 21, 23] are interesting in this respect). However I argue (lines 50-56) that the practical consequences for inference are unclear, and can only be determined by comparing the EBF to a proper Bayes factor in a manner such as done here.

Having demonstrated a systematic difference between EBF and proper Bayes factors, it remains to justify making the correction. At lines 57-58 I argue that this is to interpret EBF on the same scale as a proper Bayes factor. This is justified in turn (lines 83-86) by the development of objective units of evidence in section 4.4. This is further emphasised by new text at line 656: “The existence of such a scale is a crucial advantage of the Bayes factor, for without it there is no way to objectively interpret the strength of evidence. It is this scale that motivates adjusting the posterior Bayes factor to the EBF”.

These are basically arguments about interpretation, which is the essence of the hypothesis testing debate. A more formal justification is not really possible, beyond existing arguments for Bayes factors or WAIC.

Also, when it comes to the concrete application to the normal mean in sectison 4.1, it seems the answer is approximately 2.33 exp(-z^2/2), which essentially is a constant multiple of the standard minimum Bayes factor which is exp(-z^2/2) and very close to the minimum Bayes factor in equation 10 of Held and Ott which is approximately 2 exp(-z^2/2). Despite the numerical similarity, the proposal under review seems to suggest a dramatic difference between the calculations here and the standard results, which I do not really see. Overall, at least to me, the proposal does not make a sufficient case for the statistical approach taken here.

It is correct that in this special case, the EBF is a multiple of these minimum Bayes factors, and it is worth pointing this out. The key difference is that the EBF is exact rather than a lower bound, and can therefore support a conclusion in favour of either hypothesis. The minimum Bayes factors can only quantify an absence of evidence and thus discourage any decision making. Furthermore the EBF has also been developed here for a range of other tests beyond the scalar normal mean.

These points were previously made at line 615 but I have added a new paragraph at line 478 to stress them further. As throughout, the key advantage of these results is in interpretation.

Reviewer #2: The article proposes an empirical Bayes factor in order to obtain a measure of evidence in favor of a hypothesis to be tested. In his approach, the data are used in the construction of the priori with the justification that, although the data are used twice, the data used in the posteriori would be obtained from a replication of the experiment.
The author defends the use of his proposal, highlighting the interpretability of the proposed empirical Bayes factor.
The text presents the formulation of this proposed measure of evidence and how it is expressed in tests of some distributions, such as normal, t, binomial, F and non-parametric tests, and also in multiple tests. Furthermore, it compares for some situations the relationship between the P-value and the empirical Bayes factor. Additionally, practical applications are presented, exemplifying that the empirical Bayes factor presents adequate conclusions to the tested hypotheses. The author ends the article assuming that the proposal is not purely Bayesian, but that the objective was just to obtain a good measure of evidence in favor of a hypothesis.
In my opinion, the proposed measure is a creative alternative for calculating the evidence in favor of a hypothesis, in addition to correcting inconsistencies that can be found when using the traditional Bayes factor, without correcting the bias. In the supplementary material, the author explains how to interpret and use this measure (even without the supplementary material it is more difficult to understand how to consider and interpret the result obtained in the calculation of the proposed measure) and shows that, although there is no improvement to replace a value- P, the results can be considered a little more interpretable. The work even includes a package in R that was developed to calculate the proposed measure. My recommendation is to accept the submission.

This is an accurate summary which I appreciate. If it is difficult to understand from the paper how to interpret a result, the R library and web material provide practical guidance at a less technical level.
