# Supplementary material for: Empirical Bayes factors for common hypothesis tests
Source: PLoS One. 2024 Feb 22;19(2):e0297874. doi: 10.1371/journal.pone.0297874 (PMC10883543; doi:10.1371/journal.pone.0297874)
Supplement: Attachment — Submitted filename: Response to reviewer 3.docx [file pone.0297874.s003.docx]

# Response to reviewer 3

Thank you for the feedback. My responses are in the indented paragraphs. Line numbers refer to the change-tracked document.

Reviewer #3: - Overall: The paper was well referenced, and your main motivation and derivation for the empirical Bayes factor appears sound, and I could not see any problems with the mathematical details. My main concern is that you have oversold your method. In many places I think your claims for the empirical Bayes factors are too broad. In other words, the EBF cannot do as much as you claim it can. See my detailed comments, especially with respect to lines 60-61 and 81-83 and 87-89 and Section 2.5.

lines 60-61: you state "This allow[s] standard routine analyses to proceed with minimal change, essentially replacing the final calculation of a P-value with an EBF". This is confusing to me because the EBF is acting like a Bayes factor which is measuring something different from a P-value. P-values relate to rejecting the null hypothesis (and frequentists treat the null and alternative hypotheses in fundamentally a different manner...you cannot reject the alternative hypothesis with large p-values), Bayes factors are interpreted as in line 97 (and Bayes factors treat the hypotheses similarly, so that BF01=1/BF10). So I would delete this sentence.

I agree that EBF is not an exact replacement for P. But it is important to note that the method requires little change to routine practice. I have modified the sentence to “… routine analyses to proceed as usual, with the EBF calculated at the same stage as a $P$-value”.

Here in the Introduction I do not expand further, but I have added text elsewhere to address the different interpretations of P and BF, see below.

Line 64: you state: "the EBF is equivalent to the difference in WAIC under a vague prior". It is the difference between WAIC and what?

The wording is difference in, not difference between. The sentence is correct, since the EBF refers to two models and so must the difference in WAIC. The same grammar is used for BIC at line 468.

lines 81-83: you talk about strength of evidence for qualitatively describing Bayes factors (like Table 1 of the supplement), and then you talk about "Interpretations have been proposed with base 10...". Finally, stating "But as these scales are subjective, they do not of themselves warrant the Bayes factor interpretation." I do not understand the last sentence. All of the qualitative interpretations of Bayes factors (as in Table 1, Supplement) are subjective, but they are still a "Bayes factor interpretation", albeit an interpretation based on somewhat arbitrary descriptions.

In the preceding paragraphs (lines 66, 77-78) I imply that previous work has been limited by the lack of a Bayes factor interpretation. But I do not say why that would be desirable. This paragraph says that precise updating and subjective interpretation are weak justifications, but the 3.73 scale gives a stronger reason. I have reworded the paragraph to clarify this point (line 88); see also the next comment below.

I do not understand why your interpretation of Bayes factors with base 3.73 is much different than the interpretations of log10, natural log, or log2. It is just an argument for using base 3.73 instead of 10, e, or 2. Unless I am misunderstanding, then the real impact is another column with new rows to be added to Supplementary Table 1 with your terminology (see Suppl. Table 3) at the appropriate likelihood ratio values (as in Suppl. Table 4), for example, LR=3.73 gives 'weaker disbelief', LR=13.9 gives 'stronger disbelief', etc. Is that right? Your qualitative interpretations seem reasonable, and your derivation of the 3.73 scale is interesting, but I just do not see yours as "objective" and the previously proposed interpretations as "subjective". In my mind, all are somewhat arbitrary. Similarly, rejecting at p<=0.05 is also somewhat arbitrary and is just a convention.

The 3.73 scale differs from the others in that the qualitative levels of evidence are defined by the scale itself, not from my experience with it. While base 10 can be argued as intuitive, Jeffreys has no objective reason to suggest levels at powers of $\sqrt{10}$. Similarly, Kass & Raftery argue for the deviance scale, but have no objective reason to start at exp(1) and then go to exp(3) and exp(5). Base 2 could be justified for binary decision making, but Royall has no objective reason to choose $2^{3}$ and $2^{5}$ as the qualitative levels. In all cases it is unclear whether the levels can be extended beyond those listed in Supp Table 1.

In contrast, the rationale for 3.73 (evidence to update weaker belief to stronger belief) immediately gives one for ${3.73}^{2}$ (weaker disbelief to stronger belief), and all integer powers (Supp Table 2), unrelated to my personal experience. This is objective in a way that the other scales are not.

I think this is important because an objective Bayes factor has limited appeal if it can only be interpreted subjectively. We might as well use the posterior Bayes factor, Bernardo’s BRC etc, and refer them to our experience of “strong evidence” etc for those quantities. This is essentially what has happened with P-values, leading to the debates about “significance”.

Of course 3.73 is not the only possible scale, just as there are different kinds of objective priors. But I like it because it accords with the experience of the authors in Supp Table 1, and the P-value calibrations for EBF align with traditional significance levels in Table 6.

Nevertheless you are not obliged to accept this scale, so I have toned down the claims of objectivity: line 87 replaced “objective” with “mathematical”; lines 90, 588 and 698 removed claim of a “fully objective framework”; line 569 and supplement line 29 replaced “objective” with “appropriate for general use”.

Although I have defined weaker and stronger *belief*, I want to avoid such terms for the *evidence* as linguistic fluidity is another source of subjectivity (supp lines 36 and 89). Therefore I separate the information in Supp Tables 1 and 3, but it is not difficult to cross reference between them.

lines 87-89 (see also lines 25-26): You state "We will see that this framework yields a surprising re-interpretation of classical significance testing, realizing a compromise between Bayesian and frequentist paradigms." I think this overstates what you are doing. You have a way to estimate Bayes factors with a more objective prior, but it is still a Bayes factor, which is measuring something different from a P-value. It is not doing classical significance testing, which only rejects the null hypothesis or fails to reject the null hypothesis (since large P-values are not necessarily evidence for the alternative, they could just be a lack of evidence against the null hypothesis). For a recent Bayesian interpretation of frequentist inferences, see e.g., Fay, Proschan, Brittain, and Tiwari (2022) Statistical Science 37(4): 455-472.

The compromise is that the EBF has a fixed distribution under a point $H_{0}$ (so EBF can achieve frequentist error control), while the P-value calibrates to an EBF in which $H_{1}$ is the posterior distribution (so P can have a Bayesian interpretation). Although BF and P measure different things, the P-value is just a function of the data and so it can be used to obtain a Bayes factor if $H_{1}$ is specified. I have added text to the Discussion to expand this point, from line 649.

The “re-interpretation of classical significance testing” is given in lines 582-587: “$P=0.05$ is roughly enough evidence to decrease the qualitative belief…”.

-line 105: can you state the theta belongs in the set Theta_H, i.e., use "..for theta in Theta_H in light of x." (using the appropriate math symbols for 'theta in Theta_H').

Done at both lines 105 and 108.

-lines 204,207,210: typo? theta should be mu? For example, "H1: theta > 0" should be "H1: mu >0" ?

Thanks for spotting - corrected.

- line 218-219: "Equation (12) can therefore be used for the usual tests of contingency tables and goodness of fit." I do not understand this sentence. Usualy a goodness of fit test is calculated as a "pure" significance test (see e.g., Cox and Hinkley, 1974, Theoretical Statistics, Chapman & Hall), where the alternative hypothesis is not explicitly defined. Please either give more details or delete that sentence.

I accept this point. I have deleted the sentence and mentioned goodness of fit tests in the Discussion, line 659.

- line 226-7: you state "This formulation accommodates the classical one- and two-sample test tests for the means,...." Are these one-sided tests or tests of the null that mu=0? Please explicitly state the two hypotheses. If it is H0: mu=0 vs H1: mu ne 0, then how do you integrate over Theta_H0?

Equation 13 is a general expression for any $\Theta_{H}$. The integration uses the cumulative distribution of $t$, in practice using standard software. Similar remarks apply to section 2.4 on F-tests.

For clarity I have moved the discussion of classical tests to the end of this section, with the details about $\Theta_{H_{1}}$ added.

-line 262: You give the expected bias in Table 2 when Theta_H=[0,1]. I am not quite sure how Table 2 applies to standard hypotheses such as H0: p <= 0.5 vs H1: p > 0.5. Please give an example like that so the reader has more of an idea how it applies in practice.

Equation 16 gives the calculation for any $\Theta_{H}$. I have added the values for $H_{0}:p\leq0.5$ to Table 2, along with text at lines 276-278 noting that the bias cancels when $H_{1}:p>0.5$.

-lines 267-8: You state "This dependence on the sampling model is a departure from the likelihood principle,...". But even using Jeffreys prior with the usual Bayes factor on the binomial and negative binomial distributions is a departure from the likelihood principle, because it gives different priors for the two distributions (see e.g., Robert, 2007, The Bayesian Choice, Springer, p. 132). Please rewrite, otherwise it makes it seem like EBF is uniquely special in this sense.

I have changed “a departure from” to “a violation of” which no longer suggests a default compliance (lines 282 and 635).

- Section 2.5: One of my primary concerns with this paper is best explained with this section. Suppose you have a permutation test p-value. The p-value is calculated under exchangeability assumptions that hold under the null hypothesis but not under the alternative. For example, a Wilcoxon-Mann-Whitney test is like that. Then there is a fundamental difference between the null hypothesis (p-value is interpreted under the assumptions of the null hypothesis) and the alternative hypothesis (p-value is not interpreted under the assumptions of the alternative hypothesis). So the null and alternative hypotheses are treated differently. Small p-values reject the null but large p-values do not reject the alternative! But for Bayes factors the two hypotheses are treated similarly, since BF01 is equal to 1/BF10. So there is a fundamental difference between p-values and Bayes factors. You can talk about the relationship between the two (see e.g., Held and Ott, 2018, ref 41), but you will have to be much more careful and precise about the relationship than you have been in your paper. I am afraid readers will misunderstand the interpretation of EBF applied to nonparametric test. In fact, I cannot see how the EBF can be precisely and properly interpreted for the nonparametric P-value case. It would help me if you could go through the details of a simple example of a nonparametric p-value (e.g., Wilcoxon-Mann-Whitney test), write out the two hypotheses, and interpret the EBF (for both EBF_01 and EBF_10=1/EBF_01).

I agree that “non-parametric EBF” is inaccurate, and have replaced it with “P-value EBF”. Section 2.4 now begins: “In some situations only a $P$-value may be available, such as in non-parametric tests based on ranks. Here an EBF can be obtained by assuming that P-values arise from a Beta distribution. This assumption may not hold in reality, and such EBFs should be viewed as no more than a rough measure. However, the following result provides a useful rule of thumb.”

A parametric model (ie $Beta(1,\beta)$) is imposed on the P-value, with the alternative hypothesis as the posterior distribution $Gamma(2,-\log\left( 1-p \right))$ (line 329).

In addition to the caveat above, I have added a paragraph to the Discussion (line 655) to acknowledge the difference between non-parametric $P$-values and parametric EBFs, also including goodness-of-fit tests. At lines 321-322 and 662 I note that these EBFs should be interpreted with caution, as per your concern.

- lines 359-360: Please define H in "given that H is true for test i". Since theta_i is different for each i, I assume the H must be different for each i. If not, please explain. Please give more details.

I have modified the notation to distinguish the hypotheses on different parameters, while allowing them to describe a common set $\Theta$.

- line 411: "The test of a normal mean against..." Please give the details of the notation. If z=x/sigma, what is x (the mean?), and what is sigma (the known standard deviation?), and how does the sample size affect EBF and the P-value? Please have a sample size of n>1, since it is important to see how the EBF and P-values change with sample size.

I have clarified the notation from equation 8 in this case, ie z is the sample mean over its standard error (which reflects sample size). Properties of Z and P in terms of sample size are well known, and carry over to the EBF. Implications for consistency and sensitivity (power) have been discussed.

- line 414: you state: "There is clearly a correspondence between this EBF and the P-value." Please be kind to the reader and write out the P-value using the same notation as the EBF on line 413. How do they relate?

I have added the formula for p.

- line 415-17: you state: "..thus in contrast to P-values and minimum Bayes factors, [EBF] can give evidence for either hypothesis...". I do not understand how "Fig. 1 shows an almost linear relationship in log scale between EBF and P-value" yet somehow EBF can give evidence for either hypothesis but the P-value cannot. If you have that relationship, then you can just multiply the P-value by a constant to approximately get the EBF. Are you saying that we can extract a Bayesian interpretation from the P-value by multiplying it by a constant in this case?

The $P$-value is just a function of the data and can indeed have a Bayesian interpretation if the alternative is specified (line 650; see also Held & Ott 2018 [41] p396). And (rather nicely) this interpretation results by multiplying $P$ by a constant. Wagenmakers [49] has done something similar for Jeffreys default Bayes factors.

- line 420-421: "..a given P-value corresponds to the same EBF irrespective of sample size,.." Perhaps if you include the sample size in this section it might help the reader understand why this is so.

This should now be clear from the formulas for EBF and P.

-line 444-445: "...infinite sample sizes never occur..." That is not the point of consistency. The point of consistency is that as sample sizes get larger and larger, you get closer to the right answer. With inconsistent methods even as the sample size gets very large, it does not approach the right answer.

Although a consistent method gets closer to the right answer, we have no idea how close we actually are. So an inconsistent method might be more reliable at the current sample size. I have removed references to an infinite sample (line 461, also line 667).

-lines 655: "..existence of such as scale is crucial...without it there is no way to objectively interpret the strength of evidence" I think this statement oversells the "objectivity" of the scale. I think it is just another scale (see my earlier comments about lines 81-83).

I have reworded the sentence to say that it allows a “more objective interpretation” (line 694).

- Supplement line 42-43: you state "The following proposal relates the evidence to its effect on probability, independently of the prior odds." This seems to imply that the previous proposals (Table 1, Supplement) were not independent of the prior odds. But all of Suppl. Table 1 is for Bayes factors that are independent of the prior odds. Perhaps rewrite that sentence since your proposal is not that different from those of Suppl. Table 1.

Arguably, the adjectives in supp table 1 are only meaningful if the prior odds are 1. However, I have rewritten the sentence to clarify the difference between this scale and the others.
